# A GelMA/polydopamine hydrogel with PTH and osteogenically stimulated alveolar mucosa-derived stem cells promotes bone regeneration in MRONJ-affected wounds

**DOI:** 10.1186/s13287-025-04655-1

**Published:** 2025-09-29

**Authors:** Che-Chang Tu, Ming-Hsu Chen, Guan-Yu Lan, Yu-Ting Lin, Yu-Tse Lin, Wei-Chiu Tai, Jiashing Yu, Po-Chun Chang

**Affiliations:** 1 Department of Dentistry, School of Dentistry, College of Medicine, National Taiwan University, Taipei, Taiwan; 2 Division of Periodontics, Department of Dentistry, National Taiwan University Hospital, Taipei, Taiwan; 3 Department of Otolaryngology, Cathay General Hospital, Taipei, Taiwan; 4 Department of Chemical Engineering, College of Engineering, National Taiwan University, Taipei, Taiwan; 5 Graduate Institute of Clinical Dentistry, School of Dentistry, College of Medicine, National Taiwan University, Taipei, 100 Taiwan; 6 School of Dentistry, College of Dental Medicine, Kaohsiung Medical University, Kaohsiung, Taiwan

**Keywords:** Mesenchymal stem cell, Bisphosphonate-associated osteonecrosis of the jaw, MicroRNAs, Wound healing, Polydopamine, Parathyroid hormone

## Abstract

**Background:**

Medication-related osteonecrosis of the jaw (MRONJ) is a serious complication in patients taking bisphosphonates. This study aimed at developing a mesenchymal stem cell-based strategy to reduce the incidence of MRONJ and recover regeneration capability of MRONJ-affected wounds by using a gelatin methacryloyl/polydopamine hydrogel (GelMA/PD) to adhere alveolar mucosa-derived stem cells (AMCs) on the bone surface, with the osteogenically stimulated AMCs modulated by microRNA (miR) transfection, and the osteoanabolic environment activated by parathyroid hormone (PTH).

**Methods:**

GelMA/PD was synthesized by photo-crosslinking, and the incorporation of PD onto GelMA as well as mechanical properties were assessed. Rat AMCs were isolated, and the stemness was characterized. AMCs were osteogenically stimulated by miR transfection. Maxillary osteotomy was created in rats administrated with zoledronic acid and dexamethasone to simulate MRONJ-affected wounds, and osteotomy in rats without ZA served as healthy controls. Wounds were unfilled or filled with GelMA/PD alone, GelMA/PD with AMCs (GA), GelMA/PD with OAMCs (GO), or GelMA/PD with OAMCs and PTH (PO), and were assessed by gross observation, micro-CT imaging, histology, and immunohistochemistry for osteoblast-osteoclast coupling.

**Results:**

GelMA/PD exhibited modestly decreased compressive strength and superior adhesion strength compared with GelMA. AMCs were double positive for CD73 and CD90, showed trilineage differentiation capability, and were osteogenically stimulated by miR-218 transfection. Among MRONJ-affected wounds, soft tissue coverage was accelerated, with reduced sequestra and significantly greater bone volume in PO group (38.46 ± 10.02%) relative to unfilled group (21.81 ± 6.18%), and osteoblast-osteoclast coupling was evident in GO and PO groups. Soft tissue recovery, inflammation reduction, and matrix deposition on defect surfaces were more prominent in PO group.

**Conclusion:**

GelMA/PD loaded with PTH and microRNA-218-transfected AMCs could facilitate mucosal healing, recover the osteoblast-osteoclast coupling, and repair MRONJ-affected wounds, and might be a feasible strategy for managing MRONJ.

**Supplementary Information:**

The online version contains supplementary material available at 10.1186/s13287-025-04655-1.

## Background

Medication-related osteonecrosis of the jaw (MRONJ), as defined by the American Association of Oral and Maxillofacial Surgeons, refers to the condition of necrotic bone exposure in the maxillofacial region for more than 8 weeks in patients taking antiresorptive agents such as bisphosphonates (BP) or antiangiogenic agents without histories of radiation therapy or metastatic diseases involving the jaw [1]. A systematic review revealed that MRONJ developed in 1–3% of cancer patients treated with antiresorptive or antiangiogenic agents and in 0–0.2% of osteoporotic patients treated with antiresorptive agents [2]. Although the mechanisms leading to the onset of MRONJ are not fully understood, the suppression of osteoclastogenesis to affect bone turnover forms a major hypothesis for MRONJ induction in BP-treated patients [3]. Invasive dentoalveolar operation, specifically tooth extraction, is the most common predisposing factor to trigger MRONJ in patients taking oral BP [4]. Because inhibited mucosal healing following invasive dental procedures is a frequent clinical manifestation of MRONJ, infection and inflammation elicited by bacterial deposits on an opened dental wound also causes sequestra formation [5, 6]. To prevent the onset of MRONJ, minimally traumatic techniques and mucosal wound closure have been suggested for invasive dental operations in patients taking BP [7]. Adjunctive treatments such as hyperbaric oxygen, platelet-rich plasma, and low-level laser therapy have been proposed for preventing or treating MRONJ [8]. However, the efficiency and predictability of these therapies is yet to be identified.

Mesenchymal stem cell (MSC)-based treatment strategies have shown effectiveness in treating MRONJ in vivo, and allogenic MSCs are preferred because the viability and stemness of MSCs is impaired in BP-treated patients [9]. Oral mucosa-derived MSCs seem an ideal solution for preventing the onset of MRONJ based on their abundance and accessibility. A previous investigation revealed that alveolar mucosa-derived stem cells (AMCs) are multipotent with low antigenicity, and allogenic AMCs can promote the healing of extraction sockets and osseous repair in the craniofacial region [10]. Studies on how AMCs modulate MRONJ-affected wounds are still lacking. MicroRNAs (miRs) act as regulators of specific signaling pathways to modulate cellular functions [11], and gene modification via miR transfection to amplify osteoblastogenic potential might be considered to strengthen the regeneration capability of AMCs in MRONJ-affected wounds.

In addition to exogenous stem cells, the activation of metabolic activities on the native bone surface is also necessary for initiating the regeneration process [12]. Parathyroid hormone (PTH), a key regulator of calcium homeostasis, plays crucial roles in stimulating bone metabolism [13]. Teriparatide, a synthetic polypeptide PTH analog, exerts osteoanabolic effects, demonstrates a promotive effect on extraction socket healing, and prevents the onset of MRONJ [14, 15]. Gelatin methacryloyl (GelMA)-based hydrogel has been developed to deliver MSCs and polypeptide effectively according to its excellent biocompatibility, degradability, moldability, cell adherence, and the capability of polypeptide conjugation [16, 17]. However, because the affinity between teriparatide and substrates (e.g., bone, extracellular matrix) is weak, developing a tissue adhesive to immobilize PTH on the native bone surface should be considered. Mussel-inspired polydopamine (PD) possesses catechol groups that demonstrate adhesive capability on various substrates and binding ability with drugs [18, 19]. By oxidating dopamine in a prepolymer solution of GelMA, to activate adhesive properties, a PD-conjugated GelMA hydrogel (GelMA/PD) has recently been developed to provide an accessible tissue adhesive for immobilizing cells and signals [20].

This study aimed at developing a MSC-based strategy for treating MRONJ-affected wounds by adhering osteogenically stimulated AMCs (OAMCs) on the PTH-engineered bone surface. The hypothesis was that a GelMA/PD hydrogel loaded with PTH and OAMCs reduced the incidence of MRONJ in BP-treated animals. GelMA/PD served as a scaffold to load and improve the adherence of AMCs and PTH on the bone surface. The osteogenic potential of AMCs was stimulated by miR transfection, and miR-29a and miR-218, targeting downregulation of the inhibitor of Wnt signaling, were chosen as candidate miRs [21, 22]. PTH was used to provide an osteoanabolic environment on the bone surface, and the combination of OAMCs and PTH would further promote the osteogenicity of OAMCs. Maxillary osteotomy was performed to simulate an invasive dentoalveolar operation. The outcome could serve as a reference for the application of stem cells in preventing MRONJ and promoting osseous wound repair in BP-administrated patients.

## Methods

The Appendix provides information on the manufacturers of materials, formulations of media, antibodies, markers, reagents for flow cytometry, the conditions of real-time polymerase chain reaction (PCR), the sequence information of primers and probes, and the protocols for immunofluorescence and immunohistochemical staining.

### Ethical statement

All procedures performed on animals were approved by the Animal Care and Use Committee of National Taiwan University (protocol no. 20201244) and were conducted in accordance with ARRIVE guidelines. The procedures for gene recombination were approved by the Committee of Biological Safety of National Taiwan University Hospital.

### Fabrication and characterization of GelMA/PD

#### Manufacturing process

**Fig. 1:**
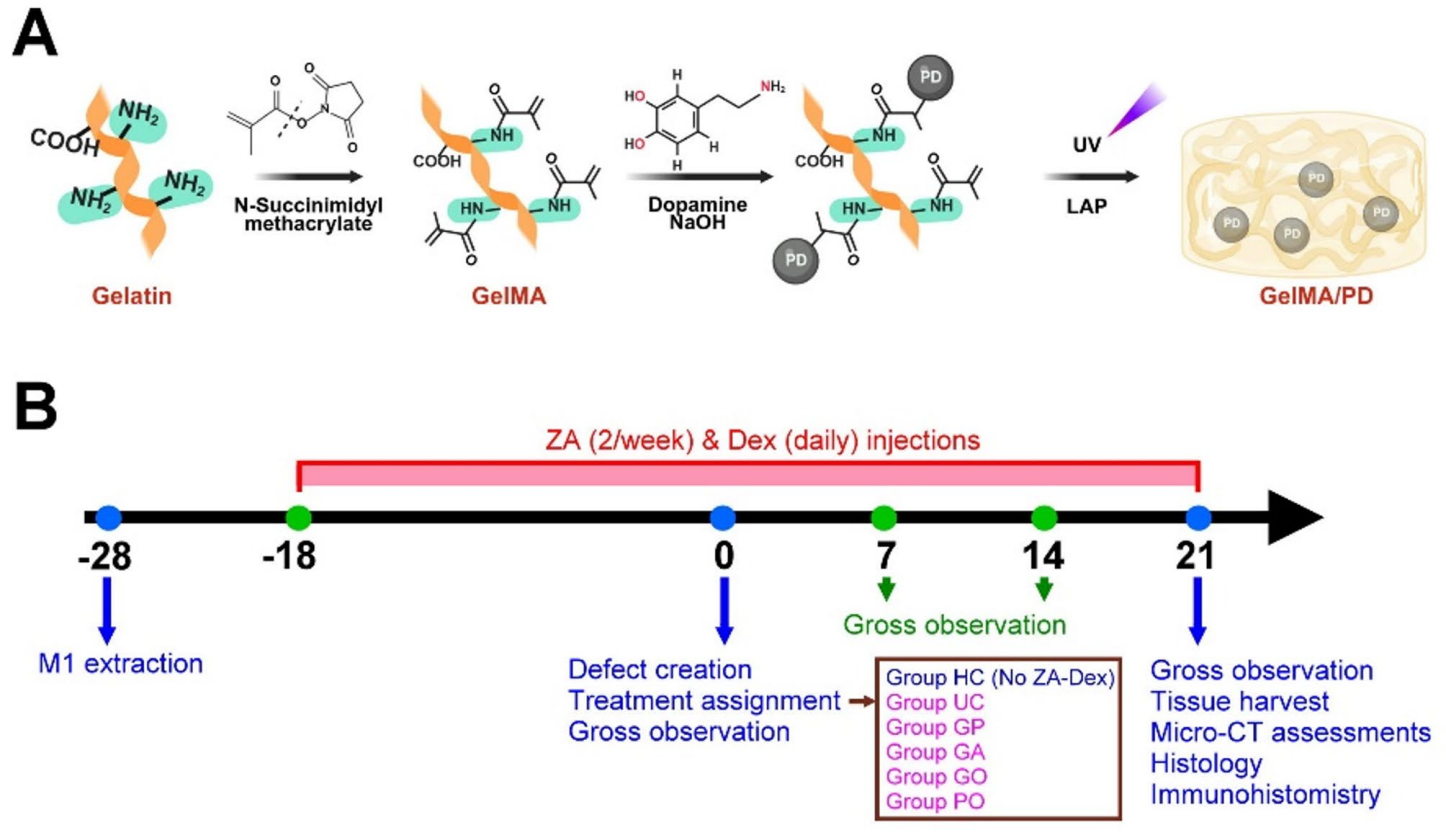
**A** The concept of GelMA/PD synthesis. **B** The timeline of animal study

The concept of GelMA/PD synthesis is illustrated in Fig. 1A. GelMA was synthesized by reacting the amine groups of gelatin with N-succinimidyl methacrylate (MA). Briefly, a 10 mg/mL MA solution was placed in a water bath, and a 20 mg/mL gelatin solution was added dropwise into it. The mixture was stirred in the dark at room temperature overnight and was then dialyzed using a 12–14 kDa dialysis membrane for 2 days. The dialyzed solution was freeze-dried and stored in a drying cabinet.

To prepare the pre-gel mixture of GelMA/PD, 14 wt% GelMA, 0.5 wt% LAP (photoinitiator), 8 wt% dopamine, and 6 wt% sodium hydroxide were mixed at a volume ratio of 10:4:5:1. The pre-gel mixtures were incubated at 37 °C for 1 h before testing to allow dopamine oxidation to form polydopamine (PD). The hydrogels were UV-cured using an ultraviolet lightbox for 10 min.

#### Fourier-transform infrared spectroscopy (FTIR) analysis

The chemical structure and bonding of samples were determined using FTIR spectroscopy. The dried sample (gelatin, MA, GelMA, PD, or GelMA/PD) was ground with potassium bromide (KBr) at a 1:150 weight ratio and pressed into a pellet. Spectra were recorded in the range of 4000–500 cm⁻¹ with a resolution of 4 cm^−1^ and being scanned 16 times.

#### Proton nuclear magnetic resonance (^1^H NMR) spectrometry

To chemically characterize the biopolymers and elucidate their molecular structure, ^1^H NMR spectrometry was performed. 10 mg PD, GelMA, GelMA with PD (GelMA-PD) and GelMA/PD were dissolved in 600 µL deuterium oxide. The ^1^H NMR spectra of these solutions were then obtained using a 500 MHz spectrometer.

#### Compression and lap shear tests

The pre-gel mixture was injected into a custom mold and exposed to UV light for complete crosslinking. To conduct the compression test, the resultant hydrogel was then compressed using an 11.94-mm compressor at a rate of 6 mm/minute, with data collected at 10 points per second.

To conduct the lap shear test, the hydrogel was applied to overlapping regions of 2 identical porcine skin specimens and UV-cured for 10 min. The specimens were pressed together, and a water-filled tank was placed on top to ensure consistent pressure. The specimens were then clamped into a tensile testing machine with a tensile speed of 1 mm/min (see the Figure in Appendix). The maximum shear force at failure was recorded, and shear strength (τ) was defined as τ = Fmax/A, where Fmax represents the maximum shear force and A is the overlapping area.

#### Viscosity and rheology

A rheometer was used to assess the viscoelastic properties during photo-crosslinking and the mechanical integrity of the cured hydrogel. The pre-gel mixture was exposed to UV light (25 mW/cm², 365 nm) while oscillating at an angular frequency of 10.0 rad/second and 1.0% strain. The storage modulus (G’) and loss modulus (G”) were recorded during the process, stopping once the storage modulus stabilized. A frequency sweep oscillatory rheology test was then performed from 0.1 to 100 rad/second at 0.1% strain.

### Isolation and characterization of AMCs

#### Cell isolation

Sprague-Dawley rats weighing 250–300 g were housed in a climate-controlled room (21 °C) on a 12:12-h light/dark cycle with free access to food and water. AMCs were harvested from 4 rats by excising 1 mm × 3 mm full-thickness alveolar mucosal tissues from the maxillary posterior region under general anesthesia (intraperitoneal injection of 0.1 mL/kg zolazepam-tiletamine and 0.5 mL/kg xylazine). After removing the epithelium, the tissues were minced, stabilized on a cell culture dish, and incubated with α-minimum essential medium, with 10% fetal bovine serum, 100 U/mL penicillin G, and 100 mg/mL streptomycin, for 10 days. All adherent cells were passaged until they achieved 90% confluence, and cells in the third passage were used for the subsequent analyses.

#### Identification of surface markers and antigenicity

AMCs (1 × 10^6^) were incubated with 0.2 mL of FcR blocker on ice in the dark for 45 min. Primary antibodies (1 µg) of CD73, CD90, CD45, CD11b, RT1A (Class I MHC), and RT1B (Class II MHC) or an isotype control were then added for 20 min. After centrifugation and washing, goat anti-mouse secondary antibodies (1 µg) were added for 20 min, and 7-aminoactinomycin D (7-AAD), a marker to identify dead cells, was then added 10 min before acquisition. A flow cytometer was used, and nonspecific protein interactions were removed according to the isotype controls. A flow cytometry analytical software was used to gate the marker-positive cells, identify a single cell population, and exclude dead cells/doublets from the forward scatter versus side scatter density plots. The frequency of marker-positive cells was tabulated from the histograms.

#### Trilineage differentiation

For osteogenic, adipogenic, and chondrogenic differentiation, 1 × 10^5^ AMCs were seeded in 24-well plates with an osteoinductive medium, an adipoinductive medium, or a chondroinductive medium. At day 14, AMCs were fixed with 10% formaldehyde for 10 min and stained with 40 mM Alizarin Red for 20 min to identify calcium deposits, stained with 60% Oil Red O for 60 min to identify oil globules, or stained with 1% Alcian Blue and washed with 3% acetic acid to identify proteoglycan deposits.

### Osteogenic stimulation of AMCs

#### Micro-RNA transduction

Synthetic mimics with GFP reporter (200 ng) of miR-29a and miR-218 were introduced into respective 100 µL competent cells by heat shock at 42 °C for 90 s and were returned to an ice bath for 1 h. Pre-warmed Luria-Bertani broth without antibiotic was added to the transformed cells, and cells were recovered by incubating and shaking at 37 °C. The transformation mixture was plated onto a Luria-Bertani agar plate containing 25 µg/mL kanamycin and was incubated at 37 °C for 16–18 h. The transformed plasmids were isolated from the colonies by using a plasmid DNA preparation kit. The success of transformation was confirmed by DNA sequencing, as described previously [23].

Transformed plasmids (0.02 µg/µL) with Lipofectamine 3000 reagent (1 µL/µg plasmid) in Opti-MEM and 3% Lipofectamine 3000 reagent in Opti-MEM were mixed at a 1:1 ratio to formulate the plasmid-lipid complex. After 15 minutes’ incubation at room temperature, the plasmid-lipid complex was added to AMCs (70–80% confluency in the 6-well plate), cultured with serum-free culture medium for 2–4 d, and returned to the culture medium containing 10% FBS and 500 µg/mL neomycin for 7 days. The success of plasmid transfection and cell viability after transfection was assessed by using immunofluorescence staining for the expression of GFP (marker of miR expression plasmid) with DAPI (conjugation with nucleus) and phalloidin (conjugation with F-actin) and was observed under a confocal microscope.

#### Osteogenicity

To identify the osteogenicity of miR transfection, unstimulated and miR-transfected AMCs were incubated in an osteoinductive medium for 10 days and were then examined using an Alizarin Red assay and quantified using an ELISA reader at a wavelength of 405 nm.

#### Gene expression profiling

The expression levels of core-binding factor alpha-1 (Cbfa1), an osteogenic gene, vasculo-endothelial growth factor (VEGF), an angiogenic gene, and collagen type I (ColA1), a marker of matrix synthesis, in AMCs were analyzed. All examined cells were seeded on 6-well dishes at 1 × 10^5^ cells/well and incubated with either ordinary growth medium (i.e., culture medium) or osteoinductive medium for 10 days. RNA was isolated using an RNA isolation kit and reversely transcribed to cDNA using a cDNA synthesis kit to perform real-time PCR, and the TaqMan probes for GAPDH (a housekeeping gene), Cbfa1, VEGF, and ColA1, were used. The level of gene expression was calculated using the comparative CT method according to the level of GAPDH and was further normalized to the expression level of unstimulated AMCs under the same culture condition. All experiments were performed in triplicate.

### Viability and osteogenicity of AMCs under PTH treatment

The miR-transfected AMCs with the most potent osteogenic potential and elevated angiogenic effect, as described in the "Isolation and characterization of AMCs", were chosen as the osteogenically stimulated AMCs (OAMC). To evaluate cellular viability under PTH treatment, AMCs and OAMCs were incubated with culture medium containing 0–500 ng/mL PTH for 24 h and were assessed using an Alamar Blue assay. To evaluate osteogenicity under PTH treatment, AMCs and OAMCs were incubated with osteoinductive medium for 7 days and then incubated with culture medium containing 0–500 ng/mL PTH for 4–8 days. The outcomes were assessed using an Alizarin Red assay.

### Preclinical validation

#### The MRONJ model and study design

A total of 30 male Sprague–Dawley rats (weighing 250 g) were used, and bilateral maxillary first molars (M1s) were extracted under general anesthesia. In 22 animals, at 10 days after M1 extraction, 50 µg/kg dexamethasone (Dex) was subcutaneously injected daily, and 1 mg/kg zoledronic acid (ZA) was subcutaneously injected twice per week, until the sacrifice of the animal. To induce a MRONJ wound, a 2.3 mm diameter, 1.0 mm deep osseous-mucosal wound was created on each healed edentulous ridge of M1 by using a dental bur without mucosal elevation at 4 weeks after M1 extraction. The wounds were randomly assigned to the following treatments: unfilled control (UC), GelMA/PD alone (GP), GelMA/PD loaded with AMCs (GA), GelMA/PD loaded with OAMCs (GO), and GelMA-PD loaded with OAMCs and 10^−6^ M PTH (PO) (*n* = 8–10/group; according to a previous study [10]). The randomization was determined by a simple draw by a blinded administrator (WCT) and was minorly adjusted to ensure that the contralateral socket received a different treatment assignment. This blinded administrator was also aware of group allocation at all stages of experiments on animals. Both AMCs and OMACs were incubated in osteoinductive medium for 7 days. 1.2 × 10^6^ harvested cells (AMCs or OAMCs) and pre-gel mixture GelMA/PD were added to 100 µl culture medium with or without PTH and were mixed thoroughly immediately before implantation. 8 animals that underwent unilateral osseous-mucosal wound creation but did not receive ZA and Dex injection comprised a healthy control (HC) group. Animals were euthanized by exposing them to carbon dioxide in the cage until complete cessation of breathing for more than 2 min at 3 weeks after the wound creation. The timeline of the preclinical experiment is illustrated in Fig. 1B.

#### Gross observation of mucosal wounds

Digital photographs of mucosal wounds were taken on days 4, 7, 14, and 21. The area and the Feret’s diameter of the remaining wound were measured using ImageJ.

#### Micro-CT assessments

The harvested maxillae were fixed in 10% buffered formalin for 3 days and then were examined using a micro-CT scanner with an effective voxel size of 18 μm. The presence of sequestra and the relative bone volume (BV/TV) within the original osseous wound was assessed using a micro-CT image analysis software.

#### Histologic and immunohistochemical assessments of mucosal wounds

The harvested maxillae were decalcified with 12.5% EDTA (pH 7.4) for 4 weeks after micro-CT examination and were then embedded in paraffin and cut into 5-mm-thick slices. For each specimen, one slice was stained with hematoxylin and eosin to demonstrate the healing dynamics, and two slices were stained immunohistochemically for the expression of bone sialoprotein (BSP), an osteoblastogenic marker, and receptor activator of nuclear factor-κB ligand (RANKL), an osteoclastogenic marker. All stained slices were observed under a light microscope, and images were acquired using a digital imaging system.

### Statistical analysis

Statistical analysis was performed using statistical software, and the analyst was blinded from the treatment assignment. One-way ANOVA with Dunnett tests were used to compare the differences among groups, and unpaired t-tests were used to compare the differences between the unstimulated AMCs and OAMCs under PTH treatment conditions. The data were expressed as the means ± standard deviation, with a *p* value of less than 0.05 considered statistically significant.

## Results

### Characterization of GelMA/PD

**Fig. 2:**
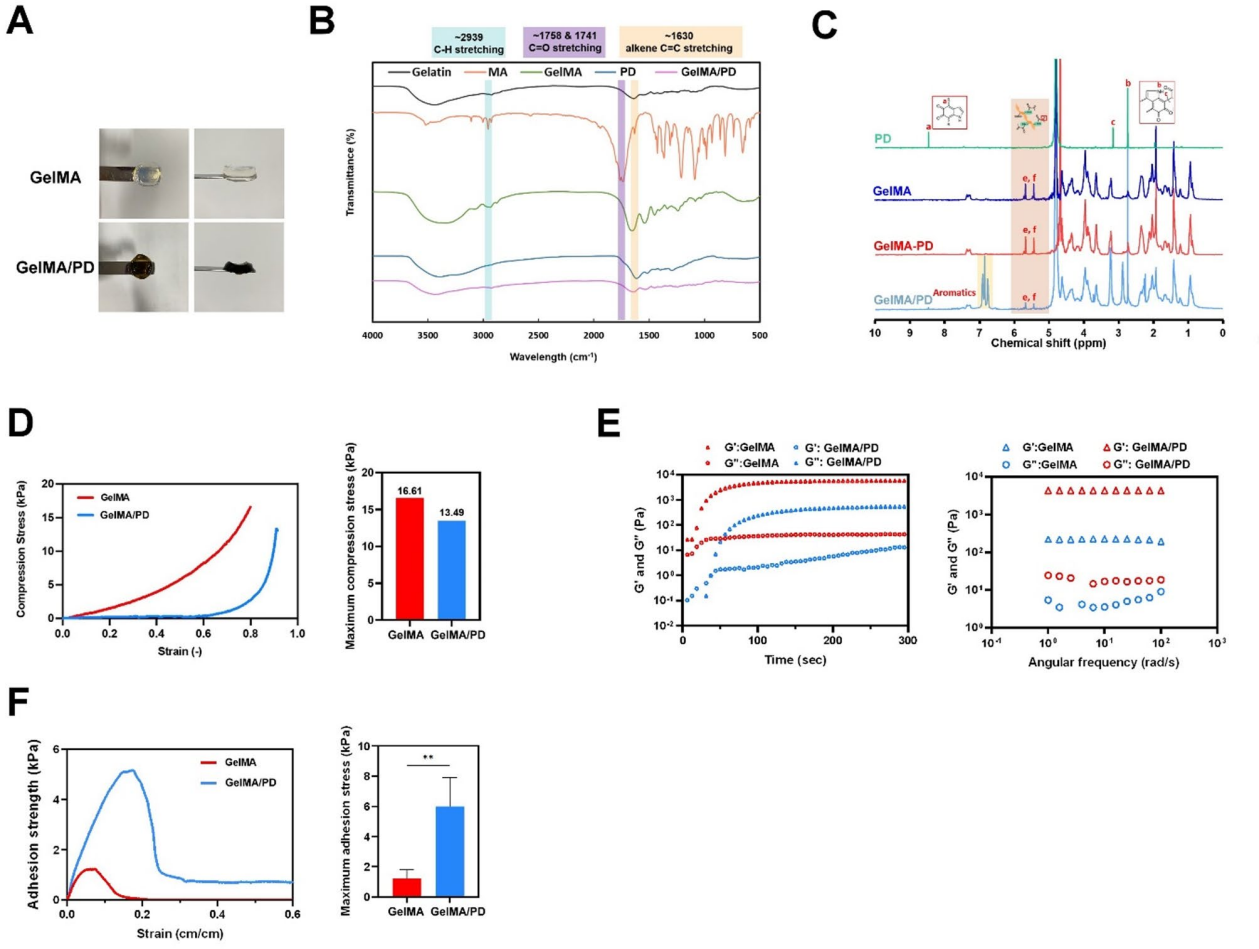
Characterization of GelMA/PD. **A** Appearance of GelMA and GelMA/PD. **B**, **C** The evidences of PD conjugation in GelMA revealed by (**B**) FTIR spectra and (**C**) ^1^H NMR spectra. **D** Outcomes of compression tests. **E** Outcomes of rheological analysis. **F** Outcomes of adhesion test. (*p < 0.05)

Both GelMA and GelMA/PD were gelated after UV irradiation for 10 min. GelMA was transparent in color, whereas GelMA/PD was brown in color (Fig. 2A). FTIR spectra of GelMA/PD showed peaks at 1631 cm⁻¹, corresponding to the C = C and C = O bonds in PD, and at 1523 cm⁻¹, corresponding to the amide group of PD. A slight shift in these peaks compared to GelMA and PD spectra revealed successful PD incorporation onto GelMA (Fig. 2B).

The molecular characterization of PD, GelMA, GelMA-PD, and GelMA/PD, was further validated through H^1^ NMR spectra (Fig. 2C). PD displayed distinct chemical shifts at 8.46, 3.16, and 2.74 ppm, which corresponded to its aromatic CH signals, secondary amine (R_2_NH), and methyl (CH_3_) groups. The olefin protons of methacrylate were identified at 5.44 and 5.68 ppm, and both GelMA-PD and GelMA/PD exhibited these peaks, confirming the successful grafting of methacrylate. The peak intensity of methacrylate diminished significantly in GelMA/PD due to the consumption of the C = C bond in GelMA during the grafting of PD, but remained consistent in GelMA-PD relative to that of GelMA. The spectrum of GelMA/PD revealed additional peaks within a broader chemical shift range of 6.70 to 6.95 ppm, which are indicative of the aromatic protons of PD.

GelMA reached a maximum stress of approximately 16.61 kPa at a strain of 0.8, whereas GelMA/PD exhibited a peak stress of 13.49 kPa at a strain of 0.9, and the compressive stress of GelMA/PD did not increase significantly until a strain of 0.6 (Fig. 2D).

GelMA exhibited a higher storage modulus (G’) than loss modulus (G”) at the outset, indicating hydrogel characteristics before complete gelation. For GelMA/PD, G’ and G” curves intersected after 40 seconds of photo-crosslinking, with G’ stabilizing after approximately 200 seconds, reflecting a slower gelation rate, and G’ was consistently lower relative to that of GelMA (Fig. 2E).

The results of lap shear test demonstrated that GelMA/PD achieved higher adhesion strength relative to GelMA, and the maximum bonding stress was significantly greater in GelMA/PD (Fig. 2F). The superior adhesion performance underscored the potential applicability of GelMA/PD as a biologic adhesive.

### Characterization of AMCs

**Fig. 3:**
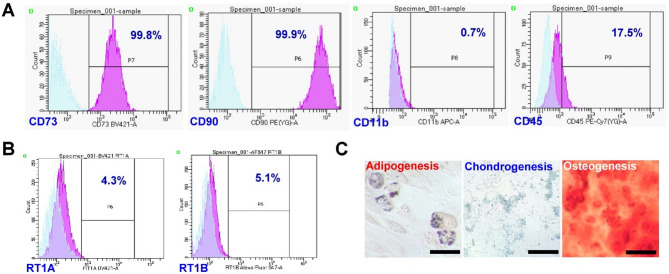
Characterization of AMCs. **A**,** B** Flow cytometric analyses of the expression of (**A**) stem cell surface markers and (**B**) histocompatibility antigens of AMCs in the third passage. **C** Trilineage differentiation of AMCs revealed by Oil Red O (adipogenesis), Alizarin Red (osteogenesis), and Alcian Blue (chondrogenesis) at day 14. Scale bar: 100 μm

Of the AMCs, > 99% were positive for CD73 and CD90, < 1% AMCs expressed CD11b, and 14.4% AMCs expressed CD45 (Fig. 3A). RT1A and RT1B were expressed in 4.3% and 5.1% of AMCs, respectively (Fig. 3B). Evident Lipid droplet accumulation, mineralized nodule deposition, or cartilage-specific proteoglycan deposition were noted in AMCs cultured with adipoinductive, chondroinductive, and osteoinductive medium for 14 days (Fig. 3C).

### Characterization of OAMCs

**Fig. 4:**
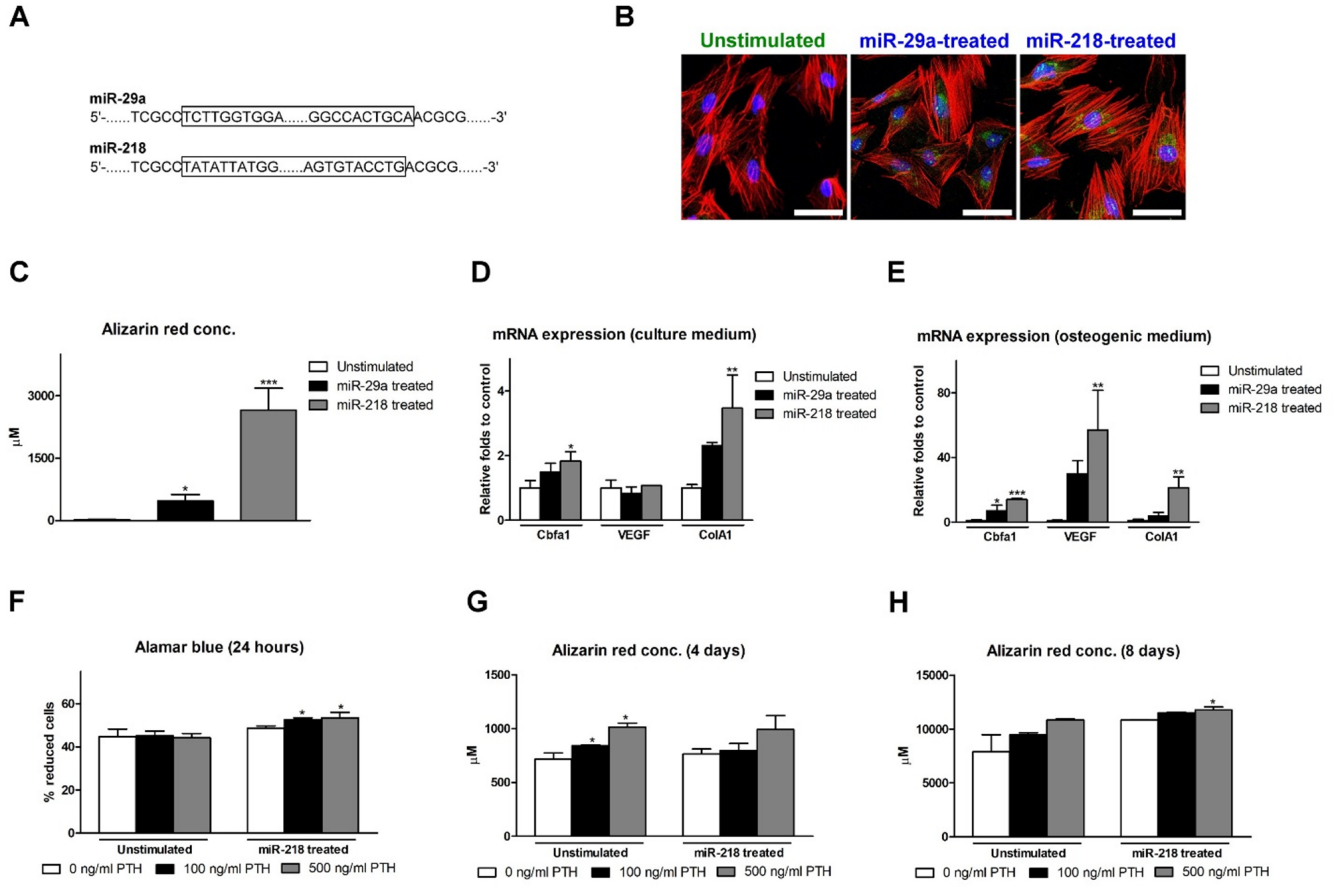
Characterization of miR-treated AMCs. **A** Full-length inserts of miR-29a and miR-218 (outlined) were successfully transformed to the competent cells. **B** The expression of miR in AMCs according the GFP reporter system. Scale bar: 50 μm. **C** Osteogenicity of miR-treated AMCs revealed by Alizarin Red. **D**–**E** Gene expression levels of miR-treated AMCs relative to unstimulated AMCs incubated with (**D**) ordinary culture medium and (**E**) osteoinductive medium at day 10. **F** Viability of miR-treated AMCs relative to unstimulated AMCs at 24 h by Alamar Blue. **G**,** H** Osteogenicity of miR-treated AMCs relative to unstimulated AMCs at (**G**) day 4 and (**H**) day 8 by Alizarin Red. (*p < 0.05, **p < 0.01)

The outcomes of DNA sequencing revealed that both miR-29a and miR-218 were successfully transformed to the competent cells (Fig. 4A). All AMCs attached well on the culture dish, and GFP signals were evidently expressed in both miR-29a- and miR-218-treated AMCs (Fig. 4B). Compared with the unstimulated AMCs, both miR-29a- and miR-218-transfected AMCs exhibited significantly higher Alizarin Red concentration, the highest of which was seen in the miR-218-transfected AMCs (Fig. 4C). When AMCs were incubated with ordinary culture medium, Cbfa1 and ColA1 were significantly up-regulated in the miR-218-transfected group (Fig. 4D). In the osteoinductive environment, the upregulation of Cbfa1 and ColA1 was further augmented, and VEGF was also significantly upregulated in the miR-218-transfected AMCs (Fig. 4E). Hence, miR-218-transfected AMCs were chosen as OAMCs.

### Effects of PTH on OAMCs

The viability of unstimulated AMCs was not apparently influenced by PTH treatment. In OAMCs, the viability was significantly improved under 100 and 500 ng/mL PTH treatment relative to that without PTH treatment (Fig. 4F). The osteogenicity of unstimulated AMCs and OAMCs were enhanced by PTH treatment dose-dependently on days 4 and 8 (Fig. 4G, H).

### GelMA/PD loaded with OAMCs and PTH for MRONJ-affected wounds

#### Gross observation and radiographic assessment

**Fig. 5:**
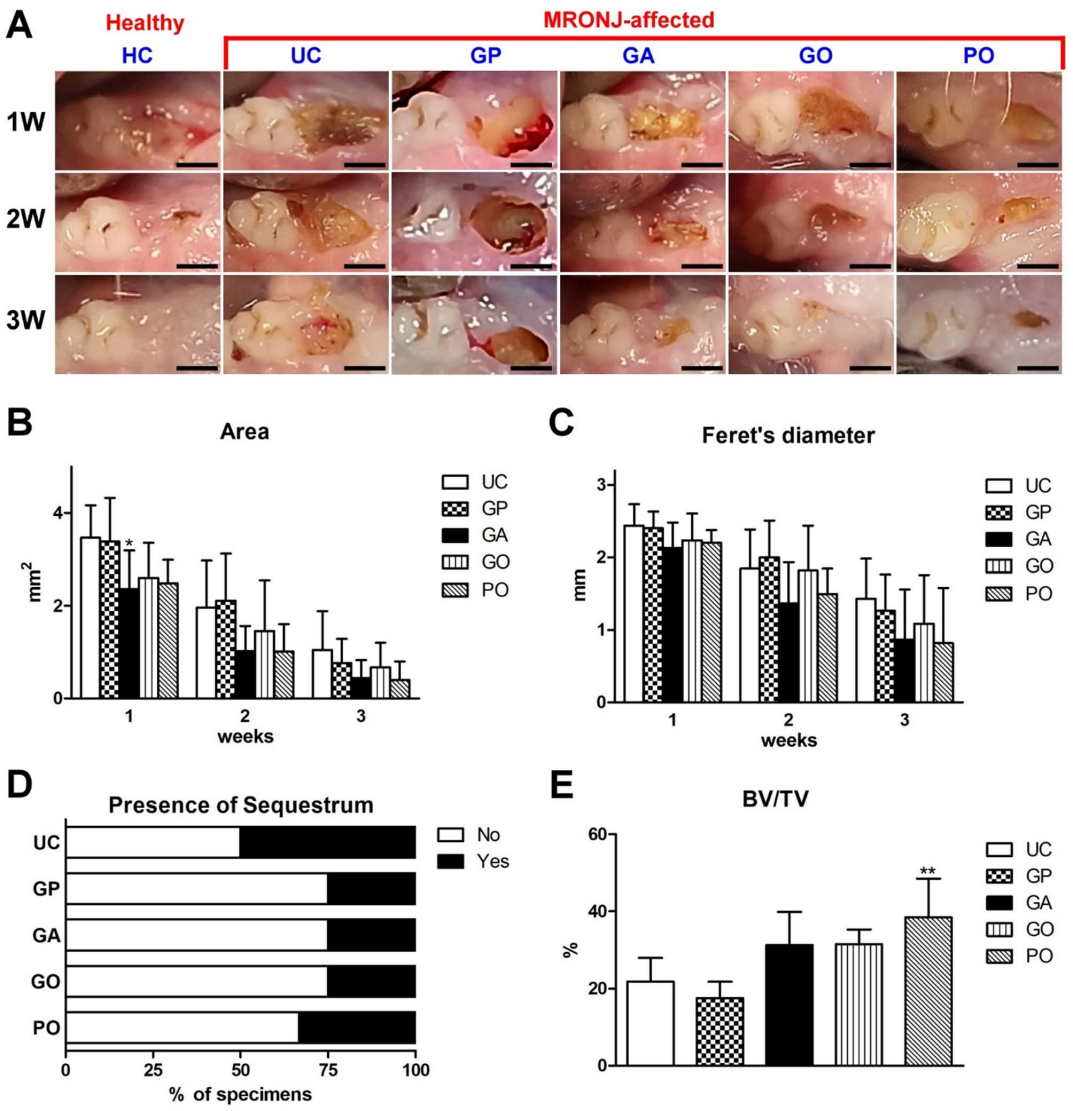
Gross observation and micro-CT analysis of MRONJ-affected wounds. **A** Representative photographs on days 7–28. Scale bar: 1 mm. **B**,** C** Quantitative analyses for the (**B**) area and (**C**) Feret’s diameter of the remaining wounds. **D** Incidence of sequestra revealed by micro-CT imaging. **E** Relative bone volume (BV/TV) in wounds without sequestra (significantly different to the UC group: *p < 0.05, **p < 0.01)

All animals exhibited uneventful healing following M1 extraction, and osseous-mucosal wounds were all created successfully after 4 weeks. In the HC group, the mucosal wounds decreased tremendously on day 7 and had completely disappeared on day 14 (Fig. 5A). In animals with BP treatment, wound healing was significantly inhibited but gradually deceased with time, but wound dehiscence was still noted in most animals on day 21. Compared with the UC group, the area and Feret’s diameter of opened wounds were smaller in the GA, GO, and PO groups at all time points (Fig. 5B, C).

The results from the micro-CT images on day 21 revealed that sequestra were frequently visible after osteotomy in the UC group, but the incidence largely decreased in the GA, GO, and PO groups (Fig. 5D). After removing the specimens with sequestra (4 in the UC group, 2 in GP, 2 in GA, 2 in GO, and 3 in PO groups), in the remaining specimens, BV/TV was significantly greater in the PO group (38.46 ± 10.02%) relative to the UC group (21.81 ± 6.18%, Fig. 5E).

#### Histologic assessment

**Fig. 6:**
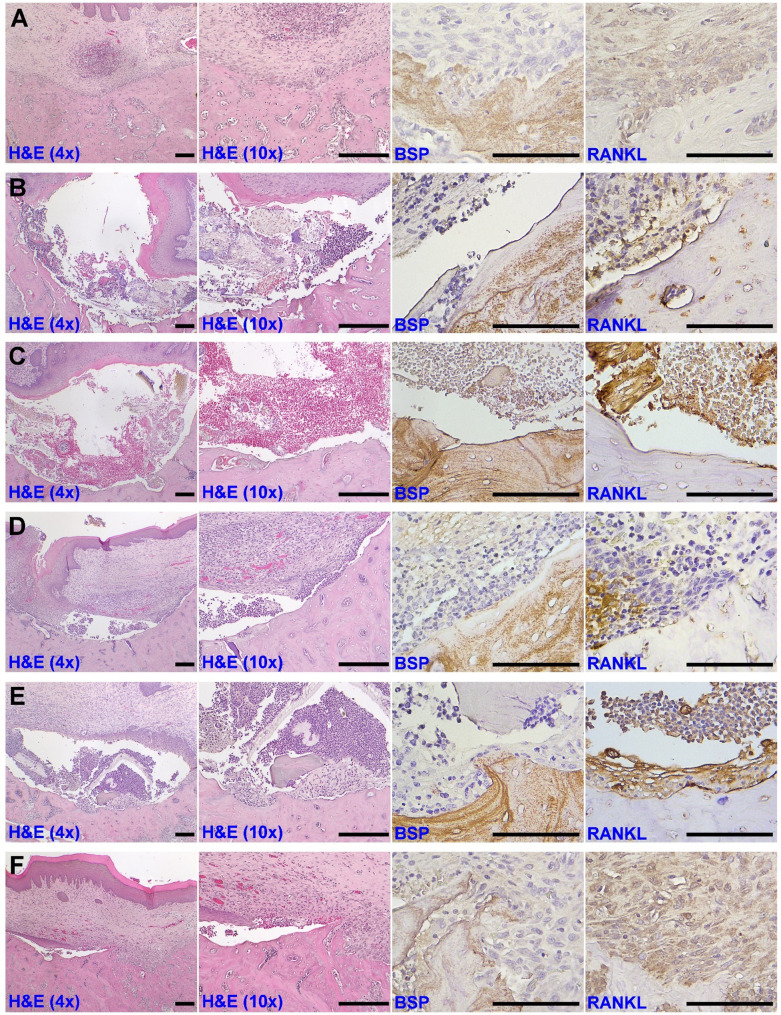
Histologic and immunohistochemical assessment of MRONJ-affected wounds on day 28. Representative images of (**A**) the HC group, (**B**) the UC group, (**C**) the GP group, (**D**) the GA group, (**E**) the GO group, and (**F**) the PO group. The images under H&E staining were under magnifications of 40x (left) and 100x (right). Scale bar: 200 μm; the immunohistochemical images (BSP and RANKL) were all under a magnification of 400x. Scale bar: 100 μm

In the HC group, the defect was completely covered by fully epithelialized mucosa and occupied by corticalized bone. Prominent expression of BSP and RANKL were noted on the bone surfaces (Fig. 6A). Sequestra or residual root fragments with inflammatory cell infiltration were noted in several animals with BP treatment (data not shown). In the UC group, mucosal dehiscence was noted in all specimens, and necrotic tissue and inflammatory cell infiltration within the wound was frequently observed (Fig. 6B). The superficial wound was nearly completely closed, but the epithelium was inversely embedded in the wound in the GP group (Fig. 6C). In the UC, GP, and GA groups, BSP was mainly deposited on the internal area of native bone, and RANKL was mainly expressed on the cells in the wounds (Figs. 6B-D). However, BSP and RANKL were barely expressed, without signs of matrix deposition, on the bone surface in both the UC and GP groups (Figs. 6B, C). Although the folding of the epithelium and inflammatory cell infiltration was still noted, matrix deposition was evident, with limited BSP and RANKL expression in the GA group (Fig. 6D). In the GO group, matrix deposition along with BSP and RANKL expression was visible on the bone surface (Fig. 6E). Furthermore, thickened epithelium with a well-organized connective tissue matrix and less inflammatory cell infiltration and prominent BSP and RANKL expression on the bone surface was noted in the PO group (Fig. 6F).

## Discussion

The incorporation of PD onto GelMA results in significant alterations to their physicochemical and mechanical properties, thereby providing substantial benefits for biomedical applications that require improved adhesion. GelMA/PD exhibited a distinct brown color with characteristic shifts in peak positions relative to those of GelMA and PD in FTIR spectra (Fig. 2A, B). The diminished intensity of the methacrylate vinyl protons with additional aromatic signals in the 6.70–6.95 ppm in GelMA/PD, as opposed to GelMA-PD mixtures, in ^1^H NMR spectra suggested the covalent incorporation of PD onto GelMA (Fig. 2C). From the mechanical perspective, the modest decrease in compressive strength of GelMA/PD relative to that of GelMA is likely due to the partial disruption of the crosslinking network caused by PD incorporation, and this hypothesis was supported by the rheological analysis (Fig. 2D, E). GelMA/PD displayed a lower storage modulus (G’) and a delayed gelation onset, consistent with the radical-scavenging, antioxidant nature of PD that interfered with photopolymerization efficiency. Furthermore, GelMA/PD demonstrated a dramatic enhancement in adhesive performance (Fig. 2F), and this superior adhesion was attributed to the catechol groups through PD grafting that established strong interfacial interaction via non-covalent binding to substrate surfaces and the coordination with metal ions [24], making GelMA/PD a potential adhesive on tissue surfaces.

The present study demonstrated that GelMA/PD loaded with OAMCs and PTH could reduce the incidence and extent of MRONJ in BP-treated animals. BPs, a class of pyrophosphates exhibiting high affinity for bone and impeding osteoclastogenesis, are widely used for preventing progressive bone resorption in patients with osteoporosis, metabolic bone diseases, myeloma, or metastatic tumors [25]. BP exposure may compromise osteoblastic behavior to inhibit bone remodeling and impede angiogenesis, therefore causing osteonecrosis, and this predicament could be deteriorated when inflammation or infection is present [1, 25]. ZA has been widely used to investigate the pathophysiology of MRONJ in animals, but the dosage and duration of ZA treatment has varied among studies [15, 26–28]. In the present study, ZA was injected biweekly, and an osteotomy without mucosal coverage was created 3 weeks after the first ZA injection. After the animals were sacrificed, wound dehiscence with significant inflammatory cell infiltration but without signs of osteogenesis, and sequestra as well as necrotic tissue were frequently observed in the UC group (Figs. 5 and 6B). These findings indicated that MRONJ-affected wounds were developed successfully in our protocol.

Because AMCs were abundant in oral mucosa and revealed the characteristics of MSCs (Fig. 3), and our previous study demonstrated AMCs could accelerate extraction socket healing and osteo-regeneration in vivo [10], AMCs were chosen as the source of MSCs in this study, although the clinical evidence of using AMCs was very limited. GelMA/PD was used as a scaffold to maximize the retention of AMCs on the exposed bone surface according to its superior adhesive performance driven by the catechol groups (Fig. 2F). The conjugation of the catechol groups also triggered a coagulation cascade [29], making GelMA/PD suitable for wound dressing. In the present study, the wound area rapidly decreased with a reduced incidence of sequestra in the GA group (Fig. 5), supporting the efficiency of AMCs for accelerating soft tissue healing and limiting bacterial contamination [10]. However, BSP and RANKL were barely expressed on the bone surface indicated that most bone-lining cells were not activated for osteoblastogenesis and osteoclastogenesis in the UC, GP, and GA groups (Figs. 6B–D). To promote osteogenicity and overcome an environment with inferior regeneration capability, AMCs were pre-stimulated via miR transfection. As shown in Fig. 2, miR-218-transfected AMCs not only contributed to greater calcium nodule formation and osteogenic gene upregulation, but they were also associated with the promotion of angiogenesis and matrix synthesis. These results support miR-218-transfected AMCs (OAMCs) as candidate MSCs for promoting osseous wound repair in BP-treated animals. The expression of BSP and RANKL, with more abundant matrix deposition on the defect surface in the GO group, indicated that signals from OAMCs partially recovered the healing potential of the bone surface, where the presence of inflammation or infection still impeded osteoregeneration in BP-treated animals (Fig. 6E).

PTH, an osteoanabolic polypeptide exhibiting pro-osteogenic, pro-osteoclastogenic, and pro-angigogenic properties, has been used to prevent or enhance the healing of MRONJ through systemic administration [15, 27]. PTH was also shown to promote the viability and osteogenicity of OAMCs in this study (Fig. 4F–H). Adhesion to the defect surface using GelMA/PD can achieve a predominant anabolic effect of PTH on the MRONJ lesion. The results from the present study showed that soft tissue healing was further promoted, and osteoblast-osteoclast coupling was recovered, with more bone formation, in the PO group (Figs. 5E and 6F). The reduction of inflammation by PTH was also reported by Castillo et al. [27], implying that PTH might play an anti-inflammatory role to prevent further destruction in MRONJ-affected wounds. Taken together, GelMA/PD loaded with OAMCs could be beneficial to recover the osteoblast-osteoclast coupling, and adding PTH further promoted osteogenesis and prevented tissue breakdown to synergistically facilitate the repair of MRONJ-affected wounds.

This study had limitations. First, the long-term functionality of the hydrogel needs further consideration, whereas this study is lack of evaluation of GelMA/PD degradation kinetics, which may significantly influence the long-term retention of therapeutic agents and scaffold integrity. The current study confirmed enhanced adhesion under controlled conditions, whether GelMA/PD can sustain these properties in vivo over time was not directly assessed. Since GelMA is enzymatically degradable, especially in protease-rich environment, the long-term performance of GelMA/PD in the dynamic oral environment remains uncertain. The oral cavity presents numerous challenges, including enzymatic activity, pH fluctuations, and microbial colonization, all of which can compromise the hydrogel’s structural integrity and adhesive properties, leading to the reduction of long-term healing efficacy in the MRONJ lesion. Further studies simulating the oral environment to determine the durability and reliability of GelMA/PD are necessary. Second, the dosing regimen of ZA may influence the severity and recovery pattern of MRONJ-affected wounds [30], and the dose equivalency between rats and humans remains unclear. Third, previous studies indicated that an intermittent low-dose of PTH could encourage osteogenesis, whereas continuous higher-dose PTH potentially led to bone resorption [30, 31], but an adequate local delivery dose of PTH to recover MRONJ has yet to be confirmed. Fourth, although 8–10 sites/group were investigated, after excluding those with sequestra or with significant histologic artifacts on the defect surfaces, specimens for BV/TV or histologic assessments were limited. Notwithstanding these limitations, this investigation still contributes a perspective to develop a MSC-based strategy for managing MRONJ-affected wounds, and further validations on large animal model and clinical trials are warranted.

## Conclusion

In MRONJ-affected wounds, mucosal healing was accelerated by using AMCs-laden GelMA/PD, and osteoblast-osteoclast coupling was recovered by miR-218 transfection on AMCs. The healing and repair of osseous defects was further promoted by adding PTH. GelMA/PD loaded with miR-218-transfected AMCs and PTH might be a feasible strategy for managing MRONJ.

## Supplementary Information

Supplementary Material 1.

## Data Availability

The data generated and analyzed in this study are available from the corresponding author on reasonable request.

## Abbreviations

GelMA: Gelatin methacryloyl
PD: Polydopamine
AMC: Alveolar mucosa-derived stem cell
OAMC: Osteogenically stimulated AMC
PTH: Parathyroid hormone
MRONJ: Medication-related osteonecrosis of the jaw
BP: Bisphosphonates
MSC: Mesenchymal stem cell
miR: MicroRNA
PCR: Polymerase chain reaction
FTIR: Fourier-transform infrared spectroscopy
^1^H NMR: Proton nuclear magnetic resonance
7-AAD: 7-aminoactinomycin D
Cbfa1: Core-binding factor alpha-1
VEGF: Vasculo-endothelial growth factor
ColA1: Collagen type I
GAPDH: Glyceraldehyde 3-phosphate dehydrogenase
M1: First molar
Dex: Dexamethasone
ZA: Zoledronic acid
BSP: Bone sialoprotein
RANKL: Receptor activator of nuclear factor-kappaB ligand
BV/TV: Relative bone volume (bone volume/tissue volume)
