# Supplementary material for: A GelMA/polydopamine hydrogel with PTH and osteogenically stimulated alveolar mucosa-derived stem cells promotes bone regeneration in MRONJ-affected wounds
Source: Stem Cell Res Ther. 2025 Sep 29;16:524. doi: 10.1186/s13287-025-04655-1 (PMC12482642; doi:10.1186/s13287-025-04655-1)

**Appendix**

Manufacturer information of materials

| Name | Manufacturer information |
| --- | --- |
| α-Minimum essential medium | Thermo Fisher Scientific Inc., Waltham, MA, USA |
| Alizarin Red | Alfa Aesar, Ward Hill, MA, USA |
| cDNA synthesis kit | Bio-Rad Laboratories, Hercules, CA, USA |
| Cell and tissue staining kit | Super Sensitive™ Polymer-HRP Detection Kit; Bio-Genex Laboratories, Fremont, CA, USA |
| Competent cells | GCI-5a; GeneCopoeia Inc., Rockville, MD, USA |
| Dopamine | Thermo Fisher Scientific Inc., Waltham, MA, USA |
| Dexamethasone | Standard Chem & Pharm Co., Ltd., Tainan City, Taiwan |
| FcR blocker | Innovex, Lincoln, RI, USA |
| Fetal bovine serum | Thermo Fisher Scientific Co., Waltham, MA, USA |
| Formaldehyde | Merck KGaA, Darmstadt, Germany |
| Kanamycin | Bioman, Taipei, Taiwan |
| Lipofectamine 3000 | Thermo Fisher Scientific Co., Waltham, MA, USA |
| Master Mix | Thermo Fisher Scientific Co., Waltham, MA, USA |
| Mimics of miR-29 | OriGene Tech., Inc., Rockville, MD, USA |
| Mimics of miR-218 | OriGene Tech., Inc., Rockville, MD, USA |
| Neomycin | InvivoGen, San Diego, CA, USA |
| Oil Red O | Alfa Aesar, Ward Hill, MA, USA |
| Parathyroid hormone (PTH) | Forteo^®^, Eli Lilly and Company, Indianapolis, IN, USA |
| Penicillin–streptomycin | Thermo Fisher Scientific Co., Waltham, MA, USA |
| Plasmid DNA preparation kit | Bioman, Taipei, Taiwan |
| Polyclonal antibody for GFP | Thermo Fisher Scientific Co., Waltham, MA, USA |
| Goat anti-rabbit IgG secondary antibody | Thermo Fisher Scientific Co., Waltham, MA, USA |
| Fluorescence conjugate | Alexa Fluor^®^ 488 conjugate; Thermo Fisher Scientific Co., Waltham, MA, USA |
| Phalloidin | Alexa Fluor^®^ 594 phalloidin; Thermo Fisher Scientific Co., Waltham, MA, USA |
| Polyclonal antibody for BSP | Thermo Fisher Scientific Co., Waltham, MA, USA |
| Polyclonal antibody for RANKL | Thermo Fisher Scientific Co., Waltham, MA, USA |
| RNA isolation kit | RNeasy Mini Kit; QIAGEN GmbH, Hilden, Germany |
| Sodium hydroxide | Honeywell Research Chemicals, Charlotte, NC, USA |
| Sprague-Dawley rat | BioLASCO Taiwan Co. Ltd., Taipei, Taiwan |
| Xylazine | Rompun 20; Bayer Animal Health, Monheim, Germany |
| Zolazepam-tiletamine | Zoletil 50; Virbac, Cedex, France |

*Materials not listed in the table were all from Sigma-Aldrich, St Louis, MO, USA.

Manufacturer information of equipment

| Name | Manufacturer information |
| --- | --- |
| Confocal microscope | LSM880; Carl Zeiss Microscopy GmbH, Munich, Germany |
| Digital imaging system | AxioCam ICc5; Carl Zeiss Microscopy GmbH, Munich, Germany |
| ELISA reader | Biotek Synergy^HT^ microplate reader; Agilent Technologies Inc., Santa Clara, CA, USA |
| Flow cytometer | BD LSRFortessa™, BD Biosciences, San Jose, CA, USA |
| Flow cytometry analytical software | BD FACSDiva™ Software, BD Biosciences, San Jose, CA, USA |
| FTIR spectroscopy | Spectrum 100, PerkinElmer, Waltham, MA, USA |
| ImageJ | NIH, Bethesda, MA, USA |
| Light microscope | Leica DM500; Leica Microsystems, Wetzlar, Germany |
| Micro-CT image analysis software | CTAn; Bruker Corp., Kontich, Belgium |
| Micro-CT scanner | SkyScan1176; Bruker Corp., Kontich, Belgium |
| Real-time PCR system | ABI 7800; Applied Biosystems, Foster, CA, USA |
| Rheometer | HR-2 system, TA Instruments, New Castle, DE, USA |
| 500 MHz spectrometer | AVIII-500, Bruker Corp., Kontich, Belgium |
| Statistical software | GraphPad Prism^®^; GraphPad Software Inc., San Diego, CA, USA |
| Tensile testing machine | TA.XTplusC Texture Analyser, Stable Micro Systems Ltd., Surrey, United Kingdom |
| Ultraviolet lightbox | UT-500UV, Univex, Jhonghe, Taiwan |

The information of the antibodies, isotype controls and markers of flow cytometry.

| Antibody | Secondary antibody | Fluorophore | Catalog No. |
| --- | --- | --- | --- |
| Surface markers | | | |
| anti-rat CD73 | anti-mouse (2Ab) for CD73 | BV421 | 551123 & 563846 |
| anti-rat CD90 |  | PE | 551401 |
| anti-rat CD45 |  | PE-Cy7 | 561588 |
| anti-rat CD11b |  | APC | 562102 |
| anti-rat RT1A |  | BV421 | 744855 |
| Anti-rat RT1B |  | Alexa Fluor® 647 | 562223 |
| Isotype antibody | | | |
| iso CD73 | anti-mouse (2Ab) for CD73 | BV421 | 557273 & 563846 |
| iso CD90 |  | PE | 550617 |
| iso CD45 |  | PE-Cy7 | 557872 |
| iso CD11b |  | APC | 562140 |
| iso RT1A |  | BV421 | 562603 |
| iso RT1B |  | Alexa Fluor® 647 | 557714 |
| Viability marker | | | |
| 7-Aminoactinomycin D |  | 7-AAD | 559925 |

*All products mentioned in the table were purchased from BD Biosciences.

Formulations of medium

| Medium | Formulation |
| --- | --- |
| Adipoinductive medium | DMEM supplemented with 10% FBS, 1% penicillin/streptomycin, 500 µM 3-isobutyl-1-methylxanthine, 1 µM dexamethasone, 10 µM insulin and 200 µM indomethacin |
| Chondroinductive medium | DMEM supplemented 1% FBS, 1% PSA, 10 ng mL^-1^ transforming growth factor beta-1, 50 mM L-ascorbic acid-2-phosphate and 6.25 mg mL^-1^ insulin |
| Osteoinductive medium | DMEM supplemented with 10% FBS, 1% penicillinestreptomycin,10 nM dexamethasone, 50 µM ascorbic acid 2-phosphate, and 10 µM β-glycerophosphate |

The conditions of realtime PCR

cDNA mixed with the Taqman probes and Master Mix to achieve a volume of 30 μl, and realtime PCR was performed using a realtime PCR system under the following conditions: hot-start enzyme activation at 95 °C for 3 minutes, followed by 40 cycles of denaturation at 95 °C for 10 seconds and 60°C for 40 seconds.

The list of Taqman probes used for realtime PCR

|  | Manufacturer | Reference Sequence | Assay ID |
| --- | --- | --- | --- |
| GAPDH | Thermo Fisher Scientific | NM_017008.4 | Rn01462662_g1 |
| Cbfa1 | Thermo Fisher Scientific | NM_001278483.1 | Rn01512298_m1 |
| ColA1 | Thermo Fisher Scientific | NM_053304.1 | Rn01463848_m1 |
| VEGF | Thermo Fisher Scientific | NM_001110333.2 | Rn01511602_m1 |

The protocol of the immunofluroscence staining

Unstimulated and miR-transfected AMCs were incubated in the culture medium for 24 hours. Cells were fixed with 3.7% formaldehyde for 10 minutes, washed by PBS several times, incubated with 1% BSA, and washed by PBS twice. Cells were incubated with rabbit polyclonal GFP antibody (1:200 dilution) in 0.1% BSA overnight, washed by PBS, incubated with goat anti-rabbit IgG secondary antibody (1:2000 dilution) and fluorescence conjugate for 45 minutes, washed by PBS, and were then stained with phalloidin and DAPI following the manufacturer instruction.

The protocol of the immunohistochemical staining

The immunohistochemical staining was performed using a cell and tissue staining kit following the manufacturer’s instruction. In brief, antigens and epitopes were unmasked by incubation with 0.05% trypsin/EDTA for 20 minutes at room temperature. To eliminate endogenous peroxidase activity, the sections were immersed in 3% H_2_O_2_ for 10 minutes. After blocking of non-specific binding with serum, the sections were incubated overnight at 4°C with the rabbit polyclonal anti-BSP (1:200 dilution), or anti-RANKL (1:200 dilution). The sections were subsequently incubated with the corresponding biotinylated secondary antibodies for 1 hour at room temperature. The color was developed by 3,3-diaminobenzidine, and sections were counterstained with hematoxylin.

Appendix Figure 1. The schematic diagram of the lap shear test.


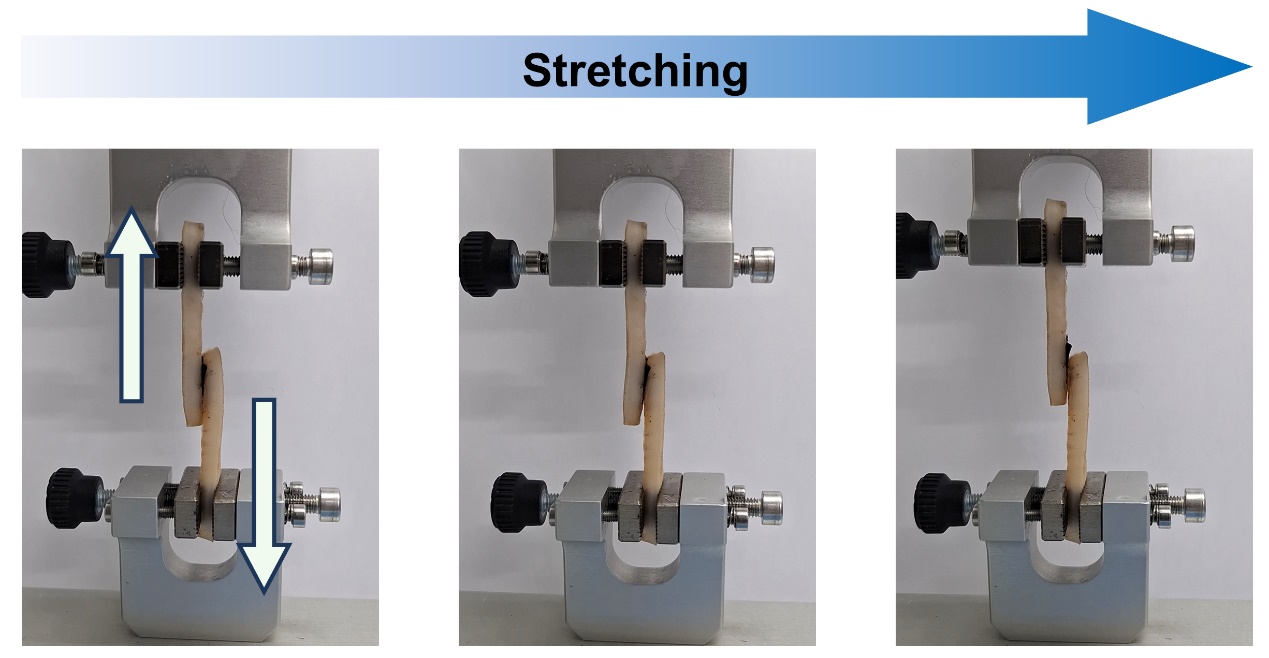

Supplement: Supplementary file 1 — Supplementary Material 1. [file 13287_2025_4655_MOESM1_ESM.docx]
